# A defect in dystrophin causes a novel porcine stress syndrome

**DOI:** 10.1186/1471-2164-13-233

**Published:** 2012-06-12

**Authors:** Dan J Nonneman, Tami Brown-Brandl, Shuna A Jones, Ralph T Wiedmann, Gary A Rohrer

**Affiliations:** 1 USDA, ARS, U.S. Meat Animal Research Center, Clay Center, Nebraska, USA

## Abstract

**Background:**

Losses of slaughter-weight pigs due to transport stress are both welfare and economic concerns to pork producers. Historically, the HAL-1843 mutation in ryanodine receptor 1 was considered responsible for most of the losses; however, DNA testing has effectively eliminated this mutation from commercial herds. We identified two sibling barrows in the USMARC swine herd that died from apparent symptoms of a stress syndrome after transport at 12 weeks of age. The symptoms included open-mouth breathing, skin discoloration, vocalization and loss of mobility.

**Results:**

We repeated the original mating along with sire-daughter matings to produce additional offspring. At 8 weeks of age, heart rate and electrocardiographs (ECG) were monitored during isoflurane anesthesia challenge (3% for 3 min). Four males from the original sire-dam mating and two males from a sire-daughter mating died after one minute of anesthesia. Animals from additional litters were identified as having a stress response, sometimes resulting in death, during regular processing and weighing. Affected animals had elevated plasma creatine phosphokinase (CPK) levels before and immediately after isoflurane challenge and cardiac arrhythmias. A pedigree containing 250 pigs, including 49 affected animals, was genotyped with the Illumina PorcineSNP60 Beadchip and only one chromosomal region, SSCX at 25.1-27.7 Mb over the dystrophin gene (*DMD*), was significantly associated with the syndrome. An arginine to tryptophan (R1958W) polymorphism in exon 41 of *DMD* was the most significant marker associated with stress susceptibility. Immunoblots of affected heart and skeletal muscle showed a dramatic reduction of dystrophin protein and histopathology of affected hearts indicated muscle fiber degeneration.

**Conclusions:**

A novel stress syndrome was characterized in pigs and the causative genetic factor most likely resides within *DMD* that results in less dystrophin protein and cardiac abnormalities that can lead to death under stressful conditions. The identification of predictive markers will allow us to determine the prevalence of this disease in commercial swine populations. This defect also provides a unique biomedical model for human cardiomyopathy associated with muscular dystrophy that may be superior to those available because of the similarities in anatomy and physiology and allow advances in gene therapies for human disease.

## Background

Losses of market-weight pigs present economic, legal and animal welfare issues to U.S. swine producers and include dead, non-ambulatory, fatigued and injured pigs [1]. Although the incidence occurs at low frequency (less than 1%) these losses are substantial due to added labor and disposal costs, along with loss of full-value product and have been estimated to be over $50,000,000 per year in the U.S. Fatigued, non-ambulatory pigs exhibit acute stress symptoms including open-mouth breathing, discoloration (red to purple) and blotching of skin, muscle tremors, abnormal vocalization and refusal to move, similar to what is observed in pigs with malignant hyperthermia due to a mutation in the ryanodine receptor 1 gene (*RYR1*) [2]. A DNA-based test has been available since the discovery of the mutation and producers have been able eliminate the unfavorable *RYR1* allele from their herds [3]. Over a decade ago it was found that half of the pigs arriving dead or dying at the packing plants carried at least one copy of the *RYR1* mutation [4]. By 2006 the number of dead and non-ambulatory pigs that carried the *RYR1* mutation was about 5% [3]; thus, the mutation was still present at a low frequency at that time [3]. However, a high proportion of *RYR1* normal pigs show a sensitivity to halothane anesthesia [5] and are more prone to becoming non-ambulatory after handling [6]. Pigs that are more sensitive to halothane exposure may also have inferior pork quality [5]. These relationships are believed to have a genetic basis; however, the specific cause has yet to be identified.

In the U.S. Meat Animal Research Center’s swine research population, a novel stress syndrome was detected. Test matings were made to characterize the syndrome’s physiological effects and its genetic basis. This study reports the identification of a novel porcine stress syndrome and mapping of the defect to dystrophin.

## Results

### Identification and challenge of stress susceptible pigs

**Figure 1:**
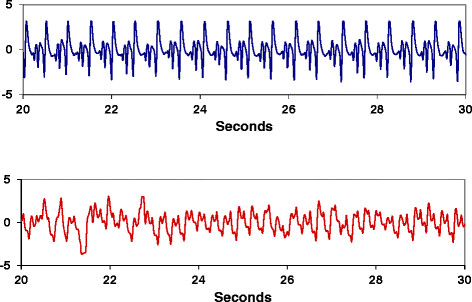
**Electrocardiograms of normal and affected littermates.** Eight week-old normal (top) and affected (bottom) littermates pigs were challenged with 3% isoflurane anesthesia and heart rates were monitored for 3 minutes. The electrocardiograms are showing traces from 20 to 30 seconds after administration of anesthesia.

The original mating that produced pigs that died after transport was repeated. Five males were born and three were classified as affected when they died after administration of isoflurane anesthesia. Because isoflurane challenge seemed to elicit a stress response, this treatment was used to test pigs from subsequent suspect litters. A total of 242 piglets from 32 litters (21 sows and 13 boars) were challenged with isoflurane anesthesia and we obtained at least one plasma creatine phosphokinase (CPK) measurement for 192 pigs, before or after anesthesia challenge. Pigs were classified as being affected if they died during handling, transport or isoflurane challenge or had high CPK levels and an abnormal ECG (Figure1). Heart rates of unaffected pigs remained steady throughout anesthesia challenge. The average CPK levels were more than three times higher in affected pigs compared to their unaffected littermates both before and after isoflurane challenge (p < 0.0001, Table1). Females that were later confirmed to be carriers, based on genotype and progeny testing, had CPK levels that were not different from unaffected animals before or after isoflurane challenge (Table1). Isoflurane treatment itself did not affect CPK levels. Of the forty-nine presumed affected pigs, based on isoflurane response, CPK levels or assessment of their ECG, only 18 died during anesthesia. These animals showed signs of respiratory distress and a rapid decline in heart rate usually within one minute of anesthesia. Eight affected animals died a few days after challenge while being transported.

**Table 1:** Plasma creatine phosphokinase activity in normal and stress-responsive pigs

**Group**^**a**^	**Normal (n = 103)**^**b**^	**Carrier (n = 37)**	**Affected (n = 35)**^**c**^
Pre-challenge	482.42 ± 41.30	573.93 ± 89.14	1635.27 ± 148.22
Post-challenge	644.37 ± 68.80	469.26 ± 51.08	1928.46 ± 136.37

^a^ Pre-challenge was at 7 weeks of age, post-challenge was at 8 weeks of age after isoflurane treatment.

^b^ Mean and standard error of plasma CPK in units/liter. Of 192 pigs that were sampled, not all the normal animals were challenged with isoflurane anesthesia.

^c^ Values were not different before or after challenge; affected pigs had significantly greater CPK activity than normal or carrier pigs (p < 0.0001).

### Mapping the stress syndrome locus

**Figure 2:**
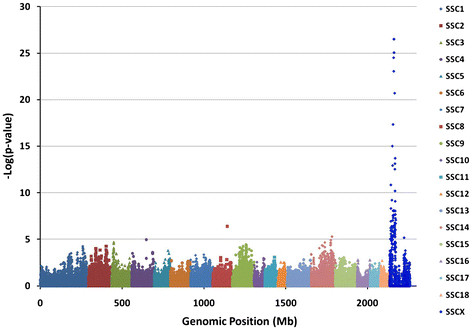
**Genome-wide case/control association for stress response in pigs.** The cumulative position in the genome (Mb) on the X-axis is plotted against the –Log_10_(p-value) for SNP associations on the Y-axis. The Bonferroni correction for genome-wide significance is a –Log_10_(p-value) of 6. The single most significant SNP associations are on the X chromosome.

To identify the genomic region associated with the stress syndrome, the coding regions of porcine orthologs of human malignant hyperthermia (MH) genes *RYR1, CACNA1S, CPT2* and *RYR2* were sequenced for SNP discovery in the proband’s family (Additional file 1: Table S1). No obvious mutations were identified comparing normal and affected siblings and the syndrome did not segregate with SNP alleles in any of the human candidate genes. A larger pedigree of 250 pigs, including 49 affected animals was then genotyped with the Illumina PorcineSNP60 Beadchip. Genotypes were called for 59,895 SNPs spanning the entire porcine genome and 49,006 of these could be mapped to build 9.2 (SGSC Sscrofa9.2/susSCR2) of the pig genome. Genome-wide association analysis was done using the case/control association option of PLINK [7]. Only one chromosomal region had highly significant SNP associations, six of which were located between 25.13 and 27.72 Mb on the X chromosome (Figure2). When only the 28 animals that died during transport or isoflurane challenge were classified as affected in the analysis, a similar but less significant association was found with the same markers at the dystrophin locus (p < 2 x 10^-11^).The two most significant SNPs on the beadchip (ALGA0099513 and ALGA0099514) are located within intron 44 of the dystrophin gene (*DMD*). All of the affected animals were hemizygous or homozygous for a shared haplotype.

### Sequencing of the porcine dystrophin gene

The exons and flanking intron sequences of *DMD* were obtained from normal and affected pigs by PCR amplification and direct sequencing. No polymorphisms were identified near splice sites. The cDNA from normal and affected animals was also amplified and sequenced and there was no evidence of alternative splicing or deletion of exons in the affected animals. The promoter regions P1, P2 and the muscle-specific promoter were sequenced and no obvious mutations were found. Forty-seven SNPs were found in *DMD* by sequencing 12 members of the affected family; of these, five non-synonymous polymorphisms were found in the coding region (Additional file 2: Table S2). One SNP in particular, 85890_783, that causes the amino acid change arginine to tryptophan at amino acid 1958 (R1958W) in exon 41 was predicted by PolyPhen-2 [8] (http://genetics.bwh.harvard.edu/pph2/) to be damaging with a probability score of 0.983.

### Association of dystrophin SNPs with porcine stress syndrome

The SNPs in the dystrophin gene identified by sequencing were genotyped across the same pedigree and analyzed for association. The 85890_783 SNP (R1958W) was as highly associated with the stress response as the two most significant SNPs on the Illumina Beadchip (Table2). The C allele (arginine) was found in all unaffected animals and the T allele (tryptophan) was found in affected and carrier females. A survey of 122 presumably unaffected 2008-born barrows from 24 sires and 76 dams all carried the C allele. The founding ancestors of this population were genotyped for the 85890_783 SNP and five of 12 Landrace boars carried the T allele, while all 12 of the Duroc boars carried the C allele. Of 122 of the founding dams (Landrace-Yorkshire composite) that were genotyped, two were homozygous and four were heterozygous for the T allele. In a panel of 192 unrelated boars of different breeds [9], the T allele was only found in 5 of 43 Landrace and 3 of 29 Hampshire boars.

**Table 2:** Associations of SNP in dystrophin with the stress response

**SNP**	**dbSNP#**	**Basepair**^**a**^	**A1**^**b**^	**F_A**^**c**^	**F_U**	**A2**	**P-value**	**-Log(p)**
84116_121	ss410758942	26546329	A	0	0.1977	G	0.000245	3.6117
84102_437	ss410758941	26635210	T	0	0.1837	C	0.001723	2.7637
85904_613	ss410758951	27322071	T	0.03509	0.1886	C	0.004925	2.3076
85890_783	ss410758971	28023949	C	0	0.7485	T	1.06E-23	22.9759
ALGA0099514	rs80929421	27689253	C	0	0.7401	T	2.19E-23	22.6605
ALGA0099513	rs80914436	27716318	C	0	0.7401	T	2.19E-23	22.6605
85896_775	ss410758960	27762149	T	0	0.1579	A	0.001687	2.7729
84024_171	ss410758936	27786935	C	0	0.1598	A	0.00143	2.8447
85894_336	ss410758939	27840323	A	0	0.4971	C	1.28E-11	10.8935
84339_200	ss410758953	27922373	G	0	0.01163	A	0.4055	0.3920
84008_345	ss410758980	27922512	A	0	0.1579	G	0.001535	2.8139
84339_333	ss410758954	27934448	A	0	0.005848	G	0.5594	0.2523
84002_411	ss410758948	27942562	C	0	0.2059	G	0.000195	3.7091
84000_82	ss410758968	28119423	G	0	0.1471	A	0.001967	2.7062
85413_679	ss410758963	28465839	C	0	0.02013	T	0.2848	0.5455
85413_571	ss410758964	28465948	A	0	0.00578	G	0.5617	0.2505
85404_372	ss410758935	28517880	-	0	0.2073	A	0.000239	3.6220

^a^Map position is based on SGSC Sscrofa build 9.2.

^b^A1 and A2 represent the different alleles.

^c^F_A is the frequency of allele 1 in cases and F_U is the frequency of A1 in unaffected.

**Figure 3:**
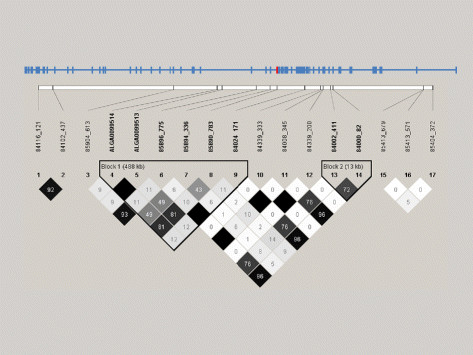
**Linkage disequilbrium plot of SNPs in the dystrophin gene in the stress family (n = 250).** The exon organization is shown above the plot, as well as the relative position of SNPs in the DMD gene. The most significant SNPs on the PorcineSNP60 beadchip (ALGA0099514 and ALGA0099513) are in complete LD with each other. The most significant SNP in the DMD gene (85890_783; marker number 8) is located in exon 41 (shown in red above).

Linkage disequilibrium (r^2^) was estimated for the SNP using Haploview 4.0 software [10] (http://www.broad.mit.edu/mpg/haploview/index.php) with the haplotype blocks based on pair-wise LD values. The calculated r^2^ values for SNP 85890_783 and the two most significant SNPs on the beadchip (ALGA0099513 and ALGA0099514) were 0.81 (Figure3) using the entire pedigree that contained the extended stress family and six other unrelated affected families. Including all founder animals, 122 barrows and 192 unrelated boars that had been genotyped for these three markers, the r^2^ value was 0.64 between SNP 85890_783 and ALGA0099513 and ALGA0099514. The two markers on the beadchip (ALGA0099513 and ALGA0099514) were in complete linkage disequilibrium in both populations (Figure3).

### Evaluation of cardiac and skeletal muscle for the dystrophin defect

**Figure 4:**
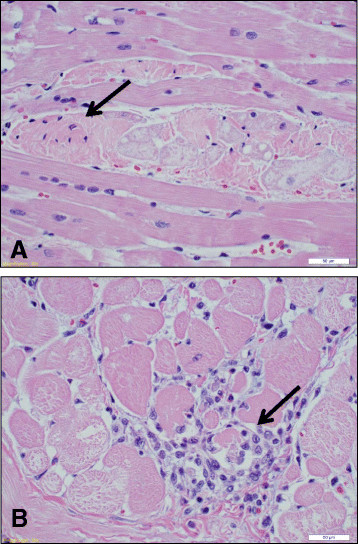
**Histopathology of left ventricular cardiac tissue from an affected pig.** Sections from a 1 year-old affected male pig show myofibrillar degeneration (magnification = 30X). **A**. Fragmentation of myocytes, pyknotic nuclei (arrow) and loss of cross-striation. **B**. Aggregation of lymphocytes and loss of myofibrils (arrow).

**Figure 5:**
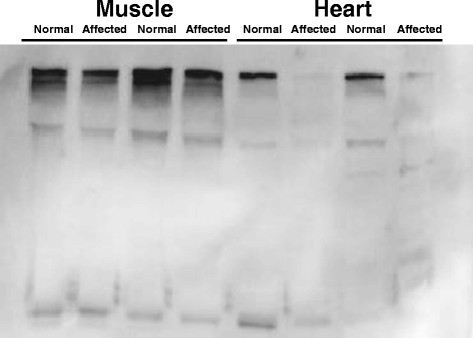
**Immunoblot for dystrophin in skeletal and cardiac muscle from 8 week-old pigs.** Pellet extract from normal and affected pigs was run in 5% SDS-PAGE and blotted with MANDYS8 monoclonal antibody which recognizes amino acids 1410–1505. Affected pigs have a reduction in dystrophin protein.

**Figure 6:**
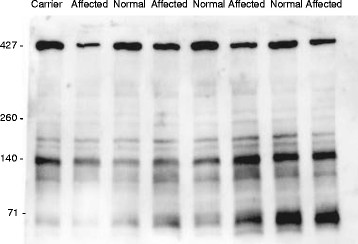
**Immunoblot for dystrophin in cardiac muscle from 6 month-old pigs.** Total protein extract from normal and affected pigs was run in 5% SDS-PAGE and blotted with MANDRA1 monoclonal antibody which recognizes amino acids 3558–3684. Affected pigs have about a 50% reduction in dystrophin protein.

Histopathology of affected left ventricular cardiac tissues showed evidence of myofibrillar degeneration and necrosis (Figure4). Myocardial fibers showed loss of cross-striation, pyknotic nuclei and associated aggregation of lymphocytes. Immunoblots of heart and skeletal muscle protein from 8 week-old pigs using MANDYS8, a mouse monoclonal anti-dystrophin antibody that recognizes residues 1431–1505 in spectrin repeat regions 10 and 11 of human dystrophin, showed a dramatic reduction (~50%) in dystrophin protein in affected pigs compared to normal littermates (Figure5). Similarly, immunoblots of heart total protein from 6 month-old pigs using MANDRA1, a mouse monoclonal anti-dystrophin antibody that recognizes residues 3667–3671 in the C-terminus of the human protein, showed a similar reduction in the amount of protein, as well (Figure6). Real-time qPCR of dystrophin in 12 affected and 12 unaffected heart tissues using primers in exons 4 and 6 showed no difference in dystrophin mRNA abundance (1.22 ± 0.313 vs. 1.42 ± 0.34 fold, p = 0.677, respectively).

## Discussion

We have identified a porcine stress syndrome that can be induced by transport, handling or isoflurane anesthesia. Affected animals quickly become non-ambulatory, have difficulty breathing and rarely recover. Isoflurane anesthesia was tested to elicit a stress response because we thought the stress syndrome was a malignant hyperthermia, similar to the classic porcine stress syndrome. Isoflurane is more readily available, less irritating to the respiratory system and has replaced halothane as a common anesthesia for veterinary applications. Although the response is delayed, compared to halothane, isoflurane can also induce malignant hyperthermia in swine [11]. Using the Illumina PorcineSNP60 Beadchip we were able to map the defect to dystrophin on the X chromosome and show that affected animals have reduced dystrophin protein in heart and skeletal muscle and elevated CPK levels in blood. These animals, however, have no apparent muscle impairment later in life. Dystrophin is the largest gene in the mammalian genome covering 2.4 megabases of DNA, and contains 79 exons and multiple tissue-specific promoters and transcripts [12]. Mutations in dystrophin cause Duchennes Muscular Dystrophy (DMD), Becker Muscular Dystrophy (BMD) and X-linked dilated cardiomyopathy (XLCM) in humans [12]. Most cases of DMD or BMD involve deletions/duplications, followed by nonsense mutations and/or microdeletions/insertions, most of which cause a frameshift or exon skipping; missense mutations are extremely rare [13-16]. DMD is usually caused by deletions, duplications or nonsense mutations that disrupt or truncate the reading frame, while BMD mutations usually preserve the reading frame [13,15]. These allelic forms are generally characterized by the severity and age of onset of the disease with DMD manifesting within the first few years of life as a progressive weakness and wasting of muscle, while BMD occurs in later decades and is more variable in severity. Patients with either DMD or BMD usually develop dilated cardiomyopathy independent of the degree of skeletal muscle involvement [17] and usually have higher than normal circulating levels of CPK [15,18]. Likewise, patients with XLCM [19,20], also have elevated CPK, probably due to increased membrane permeability [21], because the absence of dystrophin makes the membrane more susceptible to tears under the force of contraction. Cardiomyopathy that occurs without skeletal muscle involvement is frequently caused by missense mutations in the dystrophin gene [20]. Dystrophinopathies can also present with arrhythmia [22] or cardiorespiratory arrest after isoflurane anesthesia [23,24]. The pathophysiological reasons for anesthesia-induced cardiac arrest in patients with dystrophinopathies are unknown, however it is postulated that damaged, leaky muscle fibers (rhabdomyolysis) can lead to increased intracellular calcium and potassium released into the blood (hyperkalemia) and cardiac arrest [21,24]. It has been reported that isoflurane anesthesia or stress induced rhabdomyolosis, hyperkalemia and death in dystrophin-deficient cats [25]. This is the only report of stress-induced death in a dystrophic animal.

Dystrophin protein is usually absent by Western blot in DMD patients, while BMD patients usually have a decreased amount of normal or abnormal size protein [26], which is similar to the phenotype seen in the affected pigs. While it is not clear why missense mutations cause a reduction in dystrophin protein [26,27] it may be due to damage of the protein by contractile stress.

One of the nonsynonymous SNPs, (85890_783; ss410758971), which causes an amino acid change of arginine to tryptophan at amino acid 1958, was the most significant marker for the stress response and could possibly be the causative mutation. The SNP is in exon 41 of dystrophin, which codes for spectrin repeat 15 of the central coiled-coil rod domain [28]. This basic repeat region is in the second actin-binding domain of dystrophin (ABD2) [29] and has also been shown to bind lipid [30]. Although *in vitro* mutation studies have not been reported for ABD2, missense mutations in actin-binding domain 1 (ABD1) can cause protein instability, misfolding and aggregation [31,32], leading to a reduction of expressed protein.

The variant T allele of 85890_783 (Trp) could be tracked to some of the founding sires and dams in the affected families. The frequency of this allele was higher than expected, based on the rate of spontaneous deaths of feeder-aged pigs in the population. Thus, if this is the causative mutation, the penetrance of this defect is not extremely high. Further studies are required to determine the causal genetic variation, the penetrance of this phenotype and if this locus accounts for losses during transportation of market weight animals.

## Conclusions

Genetic mapping indicated the causative variation for a novel porcine stress syndrome is likely in the dystrophin gene. In addition to muscular dystrophies, mutations in human dystrophin can cause dilated cardiomyopathy, rhabdomyolosis and a malignant hyperthermia-like reaction in response to inhaled anesthesia, supporting the conclusion that this locus causes the observed phenotypes in pigs. The identification of the causative mutation in these families will allow investigation of the prevalence of this disease in commercial populations and its pleiotropic effects.

## Methods

### Animals

The animals were from a multigenerational Landrace-Duroc-Yorkshire composite population developed at the U.S. Meat Animal Research Center (USMARC) by mating Yorkshire-Landrace composite females (n = 220) to either Duroc or Landrace boars selected from the industry (n = 12 boars of each breed). This population was further developed by mating female descendants (n = 10/boar) to either a Duroc or Landrace-sired boar; Duroc-sired animals were mated to Landrace-sired animals and vice versa. Subsequent matings were random except that half-sib matings were avoided. The first affected animals identified were from the 7^th^ generation and the sire and dam were from the 6^th^ generation of this population. Litters were evaluated through generation 11. All animal procedures were reviewed and approved by the U.S. Meat Animal Research Center Animal Care and Use Committee and procedures for handling pigs complied with those specified in the Guide for the Care and Use of Agricultural Animals in Agricultural Research and Teaching [33].

### Identification of stress susceptible pigs

We identified 2 barrows in the USMARC swine herd that died after a 1 km transport to a research location at 12 weeks of age. We reproduced the original mating along with sire-daughter matings to produce additional offspring. To induce a reproducible stress response, pigs were challenged with 3% isoflurane anesthesia for 3 min at 8 weeks of age. During the course of this study, pigs from other litters were identified from their reactions to regular processing, weighing or transport to the sale barn at 5–8 weeks of age. Their littermates were also challenged with anesthesia and additional affected animals were identified. A stress response was usually indicated when the pigs stopped breathing after 1–2 minutes of anesthetic, when they were slow to awaken after anesthesia, or displayed rigidity and blotching during handling. Matings were continued with these related offspring to produce additional animals. This produced a final population of 250 animals containing 49 affected pigs for genetic analysis.

### Sample collection and phenotyping

Pigs were challenged at 8 weeks of age with isoflurane anesthesia (3% for 3 min) and body temperature, heart rate and ECG were monitored during anesthesia. During the 3 minute isoflurane challenge, ECG wave forms were obtained using a 3-wiring system recorded at 100 Hz using a 12-bit A/D. The signal was conditioned prior to recording using both a differential amplifier and a low pass filter to ensure proper signal. Blood was collected one week before challenge and immediately after isoflurane administration on most animals (n = 192). Blood was drawn into Li-heparin tubes for the measurement of creatine phosphokinase. Plasma creatine phosphokinase was measured using a 2-part reagent system (Pointe Scientific Inc., Canton, MI) in a SpectraMax M5 microplate plate reader (Molecular Devices, Sunnyvale, CA). Twenty-five μl samples were measured in duplicate and the rate of NADH formation was monitored at 340 nm and 37°C. Samples that were greater than 2500 U/L were diluted in PBS and reassayed.

### Mapping the stress susceptible locus

Initially, a pedigree of 58 pigs, including 15 affected animals, was genotyped with the Illumina PorcineSNP60 Beadchip containing 64,232 SNPs and for candidate gene SNP using the Sequenom MASSARRAY® system (Sequenom, San Diego, CA). Additional pigs were phenotyped as they were generated and subsequently genotyped. In total, 250 pigs, including 49 affected animals, their parents and unaffected siblings, were genotyped with the Illumina PorcineSNP60 Beadchip (Illumina, San Diego, CA) [34]. Genotyping of additional SNPs identified in the dystrophin gene and for candidate gene SNP was done using the Sequenom MASSARRAY® system (Sequenom, San Diego, CA). Multiplex assays with about 30 SNP/group were designed using massarray® Assay Design software. Amplicon lengths were approximately 100 bp. Reaction conditions were performed as suggested by Sequenom iPLEX chemistry. Genome-wide associations were performed using the case–control option in PLINK [7].

### Sequencing

Genomic DNA was extracted from blood buffy coats or from tails collected shortly after birth. Exons, flanking intronic regions and promoter regions of *DMD* were amplified and sequenced from twelve animals with primers (Integrated DNA Technologies, Coralville, IA) designed using Primer 3 [35] in a Dyad DNA engine (Bio-Rad, Hercules, CA) using 0.5 U Hot Star *Taq* polymerase (Qiagen, Valencia, CA); 1x supplied buffer; 1.5 mM MgCl_2_; 200 μM dNTPs; 0.8 μM each primer; and 100 ng genomic DNA in 25 μl reactions. The complete mRNA of the dystrophin muscle isoform was amplified and sequenced from cDNA using overlapping fragments and exon primers. Promoter regions were identified using Cister [36] and the cis-element matrices of TRANSFAC [37]. PCR reactions were treated with 0.1 U exonuclease (USB, Cleveland, OH), and prepared for sequencing on an ABI3730 capillary sequencer (Applied Biosystems, Foster City, CA).

### Western immunoblotting of dystrophin

Left ventricular and *longissimus lumborum* muscle samples from at least twelve affected and twelve age-matched unaffected pigs were frozen in liquid nitrogen within 10 minutes of the time of death. Protein for Western blotting was extracted as reported before [38] with the addition of protease inhibitor cocktail (Sigma Chemical Co., St. Louis, MO). Dystrophin protein was detected using 15 μg protein from cardiac and skeletal muscle run in 5% polyacrylamide gels using MANDYS8 and MANDRA1 anti-dystrophin monoclonal antibodies (Sigma Chemical Co., St. Louis, MO) diluted 1:400, goat anti-mouse IgG-HRP and SuperSignal® Western Blotting kit (Pierce, Rockford, IL). MANDYS8 recognizes residues 1431–1505 [39] and MANDRA1 recognizes residues 3667–3671 [40]. Equal loading of protein in the wells was confirmed by Coomassie Blue staining.

### Quantitative RT-PCR

Total RNA was extracted from 50 mg of porcine heart and *longissimus lumborum* muscle from 8-week old or 6-month old pigs by homogenization with TriZol reagent (Invitrogen, Carlsbad, CA) following the manufacturer’s protocol. The RNA pellets were resuspended in 100 μL DEPC treated water and absorbance was measured by spectrophotometry at 260 nm. Total RNA (2 μg) was reverse transcribed using oligo dT_18_ primer and M-MLV reverse transcriptase (Promega, Madison, WI). Primers for *DMD* (GenBank NM_001012408.1) and glyceraldehyde 3-phosphate dehydrogenase (*GAPDH*, GenBank AF017079) were designed for real-time RT-PCR using Primer 3. Primers used to amplify *DMD* were located in exons 4 and 6. Dystrophin forward primer sequence (nucleotides 423 to 444) was 5′- tgcaggtcttgcagaaaaataa -3′ and the reverse primer sequence was 5′- gcaatccagccatgatattctt -3′ (nucleotides 554 to 575). *GAPDH* forward primer sequence (nucleotides 739 to 757) was 5′-gcgtgaaccatgagaagtatga-3′ and the reverse primer sequence (nucleotides 947 to 967) was 5′-ggtagaagcagggatgatgttc-3′. Real-time PCR was performed using 1X iQ™ SYBR® Green Supermix (Bio-Rad, Hercules, CA), 0.4 ng of cDNA and 0.3 μM of each primer. The PCR reaction was performed at 95°C for 3 min followed by 40 cycles at 95°C for 20s, 58°C for 30s, and 70°C for 1 s on a MJ PTC-200 with a Chromo-4 detector (MJ Research, Watertown, MA). A quantity of 0.4 ng of cDNA was in the linear range of amplification and used for the real-time assays. The threshold cycle (Ct) for *DMD* and *GAPDH* of each sample was determined and used to calculate the fold difference between samples (n = 12 affected and 12 normal pigs) using the 2^-ΔΔCt^ method [41].

## Competing interests

The authors declare that they have no competing interests.

## Authors’ contributions

DJN and GAR designed challenge experiments, matings to generate animals, and genotyping data analysis. DJN performed biochemical experiments. TBB and SAJ were responsible for collecting physiological data and diagnosis and RTW provided bioinformatics. All authors read and approved the final manuscript.

## Supplementary Material

Additional files 1 Table S1. Human Malignant Hyperthermia Candidate Gene SNPs. Click here for file

Additional files 2 **Table S2. SNPs found in the Porcine* DMD *Gene.** Click here for file
